# Elevated DDX21 regulates c-Jun activity and rRNA processing in human breast cancers

**DOI:** 10.1186/s13058-014-0449-z

**Published:** 2014-09-28

**Authors:** Yandong Zhang, Kathleen C Baysac, Lian-Fai Yee, Anthony J Saporita, Jason D Weber

**Affiliations:** 1 ICCE Institute, Washington University School of Medicine, 660 South Euclid Avenue Campus 8069, St. Louis, 63110 MO USA; 2 Department of Internal Medicine, Division of Molecular Oncology, Siteman Cancer Center, Washington University School of Medicine, 660 South Euclid Avenue Campus 8069, St. Louis, 63110 MO USA

## Abstract

**Introduction:**

The DDX21 RNA helicase has been shown to be a nucleolar and nuclear protein involved in ribosome RNA processing and AP-1 transcription. DDX21 is highly expressed in colon cancer, lymphomas, and some breast cancers, but little is known about how DDX21 might promote tumorigenesis.

**Methods:**

Immunohistochemistry was performed on a breast cancer tissue array of 187 patients. In order to study the subcellular localization of DDX21 in both tumor tissue and tumor cell lines, indirect immunofluorescence was applied. The effect of DDX21 knockdown was measured by cellular apoptosis, rRNA processing assays, soft agar growth and mouse xenograft imaging. AP-1 transcriptional activity was analyzed with a luciferase reporter and bioluminescence imaging, as well as qRT-PCR analysis of downstream target, cyclin D1, to determine the mechanism of action for DDX21 in breast tumorigenesis.

**Results:**

Herein, we show that DDX21 is highly expressed in breast cancer tissues and established cell lines. A significant number of mammary tumor tissues and established breast cancer cell lines exhibit nuclear but not nucleolar localization of DDX21. The protein expression level of DDX21 correlates with cell proliferation rate and is markedly induced by EGF signaling. Mechanistically, DDX21 is required for the phosphorylation of c-Jun on Ser73 and DDX21 deficiency markedly reduces the transcriptional activity of AP-1. Additionally, DDX21 promotes rRNA processing in multiple breast cancer cell lines. Tumor cells expressing high levels of endogenous DDX21 undergo apoptosis after acute DDX21 knockdown, resulting in significant reduction of tumorigenicity *in vitro* and *in vivo*.

**Conclusions:**

Our findings indicate that DDX21 expression in breast cancer cells can promote AP-1 activity and rRNA processing, and thus, promote tumorigenesis by two independent mechanisms. DDX21 could serve as a marker for a subset of breast cancer patients with higher proliferation potential and may be used as a therapeutic target for a subset of breast cancer patients.

**Electronic supplementary material:**

The online version of this article (doi:10.1186/s13058-014-0449-z) contains supplementary material, which is available to authorized users.

## Introduction

Breast cancer is the second-most common cancer diagnosed in women in the United States. Multiple factors have been found to contribute to breast cancer development. Proliferation of breast cancer cells requires signals from growth factors such as estrogen and epidermal growth factor (EGF). These factors activate one of the most important transcription factors in the nucleus, activating protein-1 (AP-1), which governs the transcription of key molecules involved in cell cycle progression and survival, as well as oncogene-induced transformation and cancer cell invasion [1]-[4]. Previous studies have shown that inhibition of AP-1 activity in breast cancer cells induces cell cycle arrest and cell death as well as reduced cell invasion *in vitro* and *in vivo*[5]-[8].

The AP-1 protein family primarily consists of homodimers of Jun family members (including v-jun, c-jun, jun B, Jun D) and heterodimers of the Jun family with Fos family components (v-fos, c-fos, fosB, Fra-1, Fra2), or Jun family with activating transcription factors (ATF2, ATF3, B-ATF) [2],[4]. Jun-Jun and Jun-Fos dimers preferentially bind to the phorbol ester tumor promoter response element (TRE) while Jun-ATF dimers prefer to bind to c-AMP-responsive element (CRE) [9]. Jun/AP-1 can influence the transcription of a plethora of proteins involved in proliferation, apoptosis, invasion and tumorigenesis [3]. By itself, c-Jun can transform fibroblast cells and has been classified as a proto-oncogene, albeit a weak one [10],[11]. A strong synergistic effect occurs between AP-1 and *Ras*^*V12*^ alleles in cell transformation and tumorigenesis [10],[12]. An upstream mitogen-activated protein (MAP) kinase pathway that activates Jun N-terminal kinase (JNK) can activate c-Jun. JNK phosphorylates c-Jun on Ser63 and Ser73 [13],[14], although phosphorylation on Ser73 of c-Jun plays a more critical role than Ser63 in its activation [15] .

The DDX21 DEAD box RNA helicase has been recognized as an important nucleolar protein involved in ribosome RNA processing as previous groups have found that depletion of DDX21 results in significant reduction of 18S and 28S rRNA levels in numerous cell types [16]-[18] and DDX21 has been found to associate with 45S and 32S rRNA species [18]. DDX21 mRNA expression has been correlated with disease-free survival in breast cancer patients [19] and accumulation of DDX21 has been observed in colon cancers and lymphomas [20],[21]. DDX21 has also been shown to interact with c-Jun and has been implicated in c-Jun-mediated cellular differentiation [22]. Knockdown of c-Jun causes a diffusion of exclusively nucleolar DDX21 to partially nuclear localization [18].

In this report, we found that DDX21 is highly expressed in breast cancer tissues compared to normal breast tissue and its expression is pivotal to maintain enhanced breast cancer cell proliferation and growth. Surprisingly, a significant number of breast cancer tissues and breast cancer cell lines show nuclear localization of DDX21 protein. In cells expressing high levels of c-Jun, such as MDA-MB-231 cells, DDX21 associated with c-Jun, was required for c-Jun phosphorylation, and was essential for endogenous AP-1 activity. Moreover, DDX21 helicase activity was required to enhance the oncogenic activity of Ras^V12^, suggesting that DDX21 activities might provide requisite functions during cellular transformation. Our results demonstrate that DDX21 is an important growth and proliferation modifier that regulates oncogene-induced mammary tumorigenesis, and implicate its potential therapeutic value in breast cancers.

## Material and methods

### Cell culture

MCF-7, MDA-MB-231, SKBR3, MDA-MB-361, MDA-MB-468, CAMA-1, and BT549 breast cancer cells were cultured in Dulbecco's modified Eagle's medium (DMEM) supplemented with 10% fetal bovine serum (FBS) and penicillin-streptomycin. HCC70, HCC712 (obtained from Dr. Matthew Ellis, Washington University), HCC1428, HCC1806, ZR751, and T47D breast cancer cells were cultured in complete RPMI media supplemented with 10% FBS and penicillin-streptomycin. All cells were maintained at 37°C in 5% CO_2_. All cell lines were purchased from American Type Culture Collection (ATCC) unless otherwise noted.

### Antibodies

Antibodies were obtained from Bethyl Laboratories, Montgomery, TX, USA: anti-glyceraldehyde 3-phosphate dehydrogenase (GAPDH), anti-DDX21; Cell Signaling, Danvers, MA, USA: anti-p53, anti-cyclin D1, anti-c-jun, anti-phospho-c-jun (S73), anti-phospho-c-jun (S63); and Santa Cruz Biotechnology, Dallas, TX, USA: anti-epithelial growth factor receptor (EGFR), anti-tubulin, anti-Ras.

### Immunoprecipitation and western blots

Cells were lysed in buffer containing 1% NP-40, 50 mM Tris, pH 7.5, 150 mM NaCl, and supplemented with protease and phosphatase inhibitor cocktails (Sigma-Aldrich, St Louis, MO, USA). After incubation on ice for 10 minutes, cell lysates were sonicated to ensure complete disruption. Lysates were then centrifuged for 10 minutes at 13,000 rpm and supernatants were subjected to protein quantification assay. For western blots, 50 μg of cell lysate was loaded on a pre-cast mini-gel (Bio-Rad, Hercules, CA, USA), followed by transferring to polyvinylidene fluoride (PVDF) membrane (Merck Millipore, Billerica, MA, USA). For immunoprecipitation, cell lysates were diluted to 1 mg/ml with lysis buffer; 500 μg of total cell lysate was incubated with 2 μg of indicated antibody for 2 hours at 4°C, followed by addition of protein A/G Sepharose beads and further incubated for 1 hour at 4°C. After centrifugation at 1,500 rpm for 2 minutes, beads were washed three times with cell lysis buffer before analysis.

### Plasmids

Full-length coding sequence for DDX21 was cloned from early passage human BJ fibroblast total cDNA. Primers (forward primer: TAGTACTCGAGCCACCA TGCCGGGAAAACTCCGTAGT; reverse primer: CATGTGGATCCTGAC TTGTCATCGTCATCCTTGTAATCTTGACCAAATGCTTTACTGAA) were used to clone the open reading frame (ORF) of DDX21 into pLVX lentiviral vector at HindIII/ BamHI sites. Quick-change site-directed mutagenesis kit (Stratagene, La Jolla, CA, USA) was used to carry out the mutation K236R. Primers (forward primer: 5'-CGGACAGGAACTGGGAGGACATT CTCCTTTGCC-3'; reverse primer: 5'-GGCAAAGGAGAATGTCCTCCCAG TTCCTGTCCG-3') were designed using free software from Stratagene. Five different pLKO.1 plasmids containing short hairpin RNA (shRNA) to target each of DDX21 were obtained from the Genome Institute at Washington University. A pLKO.1 scrambled shRNA control plasmid was purchased from Addgene, Cambridge, MA, USA.

### Quantitative RT-PCR

Total RNA was extracted by NucleoSpin II (Clontech, Mountain View, CA, USA) RNA isolation kit and was converted into cDNA by SuperScript III first-strand synthesis kit (Invitrogen, Carlsbad, CA, USA). Primers were all designed by Primer Express 2.0 software and purchased from Integrated DNA Technologies, Coralville, IA, USA. The following primers were used in quantitative reverse-transcriptase-polymerase chain reaction (qRT-PCR): for cyclin D1, forward primer: 5'-AAGCTGTGCATCTACACCGA-3'; reverse primer: 5'-CTTGAGCTTGTTCACCAGGA-3'. For GAPDH, forward primer: 5'-CCCACTCCTCCACCTTTGAC-3'; reverse primer: 5'-CATACCAGGAAATGAGCTTGACAA-3'. For all qPCR analysis, SYBR green mix (Bio-Rad) was utilized.

### [methyl-3H]-methionine labeling of rRNA

Equal numbers of cells were subjected to starvation in methionine-free media containing 10% dialyzed FBS for 30 minutes. Cells were then treated with 50 μCi/mL [methyl-^3^H]-methionine and chased in complete media containing an excess of unlabeled methionine (10 μM) for the indicated times. Samples were lysed in RNASolv reagent (Omega Bio-Tek, Norcross, GA, USA) and extracted RNA was separated on agarose-formaldehyde gels and transferred to a Hybond XL membrane (GE Healthcare, Little Chalfont, UK). The membrane was cross-linked and sprayed with En3Hance (Perkin-Elmer, Waltham, MA, USA) prior to autoradiography.

### Immunofluorescence and microscopy

Cells were fixed with 10% formalin/10% methanol. Cells were then incubated with rabbit anti-DDX21 (Bethyl Laboratories) at a 1:200 dilution. Goat anti-rabbit rhodamine was applied to facilitate the visualization of DDX21 protein. To mark cell nucleoli, mouse anti-nucleophosmin (NPM) or anti-upstream binding factor (UBF) was used at a dilution of 1:100, followed by incubation with goat anti-mouse fluorescein isothiocyanate (FITC).

### Apoptosis analysis

Apoptosis assays were performed with Vybrant apoptosis kit #2 (Molecular Probes, Eugene, OR, USA) according to the manufacturer's protocol.

### Soft agar assay

1.0 × 10^4^ cells were mixed in 4.0 mL 0.3% agar/DMEM/10% FBS as the top agar and plated into 60 mm plates with 4.0 mL 0.6% agar/DMEM/10% FBS as the base agar. Plates were incubated at 37°C, checked every three days, and fed with 2.0 ml 0.3% agar/ DMEM/10% FBS every week. Colonies were photographed and counted two to three weeks later.

### Cell cycle analysis

Cells were trypsinized and washed with phosphate-buffered saline (PBS). Cells were then resuspended in PBS and 100% ethanol was added drop-wise to obtain a final ethanol concentration of 75%. Cells were centrifuged at 2,000 rpm at 4°C for 2 minutes. Cells were then washed with PBS and resuspended in PI working solution (PBS containing 1% FBS, 250 μg/ml of RNase A, 30 μg/ml of propidium iodide). Cells were filtered through a 35 μM strainer cap (Becton Dickinson, Franklin Lakes, NJ, USA) before being subjected to fluorescence-activated cell sorting (FACS) analysis.

### Immunohistochemistry

Human tumor tissue microarrays were purchased from CYBRDI (CC08-11-007). This tissue microarray contains self-paired tumor and adjacent normal tissues from 50 cases of stage I to stage III invasive ductal carcinoma. A rabbit polyclonal anti-human DDX21 antibody (Bethyl Laboratories) was used at 1:25. Tissue slides were deparaffinized in xylene and rehydrated in a series of graded alcohols and the antigen was retrieved in 0.01 M sodium citrate buffer (pH 6.0) using a steamer. The sections were then treated with 1% hydrogen peroxide in methanol for 30 minutes to exhaust endogenous peroxidase activity. After a 1 h preincubation in 10% normal FBS to prevent nonspecific staining, the samples were incubated with primary antibody at room temperature for 2 hours. Standard protocol was then followed based on DAKO (Glostrup, Denmark) Envision kit using polymer to amplify signals. A separate breast tissue microarray was purchased from US Biomax, Rockville, MD, USA (BR1503). This tissue microarray contains duplicate cores for three cases of normal tissues, three fibroadenoma, two cystosarcoma phyllodes, seven intraductal carcinomas and sixty invasive ductal carcinomas. Tissue slides were prepared as above. Rabbit anti-DDX21 was applied at 1:25 dilution onto the tissue slide and incubated at room temperature for 2 hours with humidity. After PBS washes, secondary goat anti-rabbit Alexa Fluor 594 was used at a dilution of 1:200 for half an hour at room temperature. After PBS washes, slow-fade 4',6-diamidino-2-phenylindole (DAPI) with mounting media was applied and each tissue slide was checked under fluorescence microscope and images were taken. Both immunoreactive intensity and percentage of stained cells in different areas of the same slide were analyzed according to criteria described previously [23]. DDX21 expression was designated as 0 = no staining; 1 = weak; 2 = Moderate; and 3 = strong. Additional points were scored as one, two, or three when the percentage of positive cells was less than 25%, 25% to 50%, or greater than 50%, respectively.

### Bioluminescence imaging

Nonobese diabetic/severe combined immunodeficient (NOD/SCID) female mice were purchased from Charles River Laboratories International, Inc., Wilmington, MA, USA and received standard institutional care. They were five weeks old at the time of surgery. MDA-MB-231 cells stably expressing luciferase reporter (a kind gift from Dr. Marc Diamond, Washington University in St. Louis) were infected with lentiviruses encoding shScrambled RNA or shRNA-DDX21. Two days postinfection, cells were trypsinized, washed twice in PBS, counted, and kept on ice prior to injection. Mice were anesthetized and an inverted Y incision was made between the fourth and fifth sets of nipples to expose the fourth mammary fat pads bilaterally. 5 × 10^4^ MDA-MB-231 cells were implanted into the fourth mammary glands. The final volume was 50 μl per mammary gland. The incision was sutured and mice were monitored for tumor growth at the indicated time points via bioluminescence imaging. For bioluminescence imaging of live animals, previously described mice were injected intraperitoneally with 150 μg/g D-luciferin (Biosynth, Naperville, IL, USA) in PBS, anesthetized with 2.5% isoflurane, and imaged with a charge-coupled device (CCD) camera-based bioluminescence imaging system (IVIS 100; Caliper, Hopkinton, MA, USA; exposure time 10 to 300 seconds, binning 8, field of view 12, f/stop 1, open filter). Signal was displayed as photons/sec/cm2/sr. Regions of interest (ROI) were defined manually around the abdomen, throat, and whole body using Living Image and IgorPro Software (Version 2.50) (Wavemetrics, Portland, OR, USA).

### Mouse xenograft model

NUDE female mice were purchased from Charles River Laboratories International, Inc. and received standard institutional care. They were five weeks old at the time of surgery. For NUDE mice injection, alternate reading frame (*Arf*)−/−Mouse embryonic fibroblasts (MEFs) were infected as indicated. Fibroblasts were trypsinized and re-suspended in PBS at a concentration of 1 × 10^7^ cells/ml. Five-week-old NUDE mice were injected subcutaneously with 1 × 10^6^ cells along their flank, with sample sizes of five mice per condition. Four weeks postinfection, tumors were dissected, photographed and weighed.

### Animal approval

The Animal Studies Committee of Washington University has approved the use of animals in conjunction with this study through Animal Welfare Assurance # A-3381-01 and Approval # 20130226 granted on November 18, 2013. All mouse experiments followed the guidelines of the USDA Animal Welfare Act and the Public Health Service Policy on the use of animals in research.

## Results

### DDX21 is highly expressed in breast carcinoma

**Figure 1:**
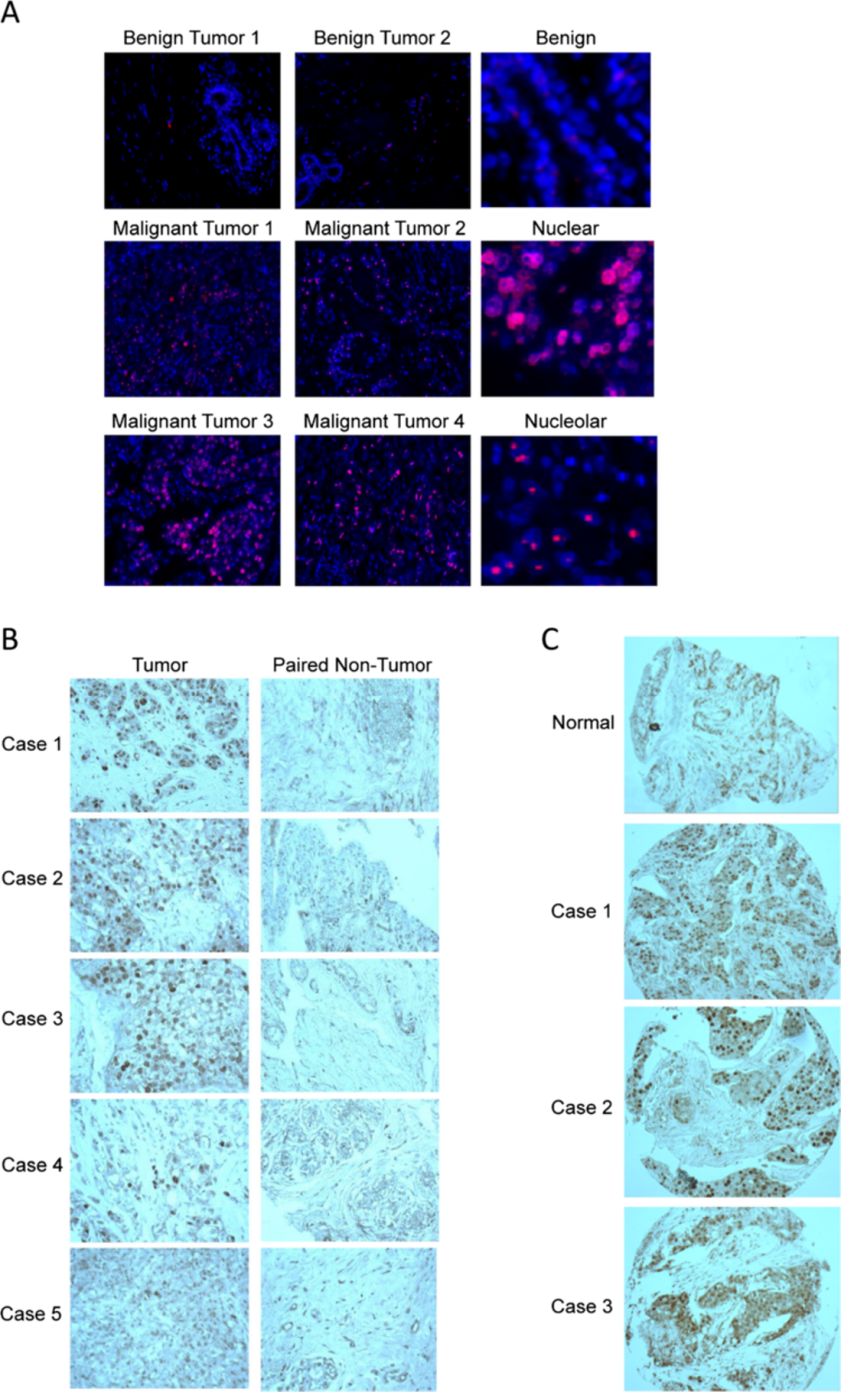
**DDX21 is overexpressed in breast carcinoma tissues. (A)** Immunofluorescence was performed on a human tissue array including 67 cases of malignant breast cancer tissue specimen. Red color depicts DDX21 expression. Blue color depicts DAPI-stained nuclei. Typical images of benign and malignant breast cancer tissues are shown. Left images were cropped from a larger field, while rights images were cropped from a smaller field. All images were taken at 40X magnification. **(B)** Immunohistochemistry was performed on a human tissue array including 50 cases of invasive ductal carcinoma with adjacent normal tissue samples. Brown color indicated positive DDX21 expression tissues. **(C)** Immunohistochemistry was performed on a human tissue array including 70 cases of various stages of breast carcinoma. Typical images of positive-stained samples are shown. DAPI, 4',6-diamidino-2-phenylindole.

We performed immunohistochemistry staining for DDX21 with several sets of human breast cancer tissue samples to determine whether DDX21 overexpression occurs in breast cancer. First, we performed immunofluorescence staining for DDX21 with a breast tumor tissue array including 67 cases of various stages and subtypes of human breast cancer. Our results indicated that DDX21 was highly expressed in 25% of the 67 cases of breast tumor tissues, while normal breast tissue and benign tumors all exhibited very weak staining for DDX21. Representative images of these tumor tissues are shown in Figure 1A. Estrogen receptor (ER), progesterone receptor (PR), human epidermal growth factor receptor 2 (HER2) status and DDX21 scores are listed in Additional file 1: Table S1. DDX21 staining was primarily observed in nuclei or in nucleolar structures (Figure 1A). Strong DDX21 staining correlated with a high rate of proliferation as detected by Ki67 on these tumor tissues (Additional file 1: Table S1). However, not all of Ki67-positive breast cancer tissues displayed strong DDX21 staining, suggesting that DDX21 is not simply a marker of proliferation. ER and PR status showed no correlation with DDX21 expression levels, while DDX21 appeared to be highly expressed in a majority of HER2-positive breast cancers (Additional file 1: Table S1). In addition, we also performed a second tissue array with 50 pairs of nontumor and tumor tissues from unique breast cancer patients (grade I to grade III). Nearly 20% of breast tumor tissues exhibited stronger immunostaining of DDX21 than the paired normal breast tissue in grade I to grade III breast carcinomas (Figure 1B). Similarly, immunostaining of a third breast cancer tissue array showed that DDX21 was highly expressed in 16 out of 70 cases (Figure 1C). Additionally, The Cancer Genome Atlas (TCGA) datasets from recently sequenced human basal breast cancer samples showed overexpression of DDX21 mRNA in 14/81 (17%) samples (cBioPortal). Taken together, DDX21 is highly expressed in a significant portion of breast cancer tissues where it localizes to either nuclei or nucleoli.

### Nuclear and nucleolar DDX21 is highly expressed in established breast cancer cell lines

**Figure 2:**
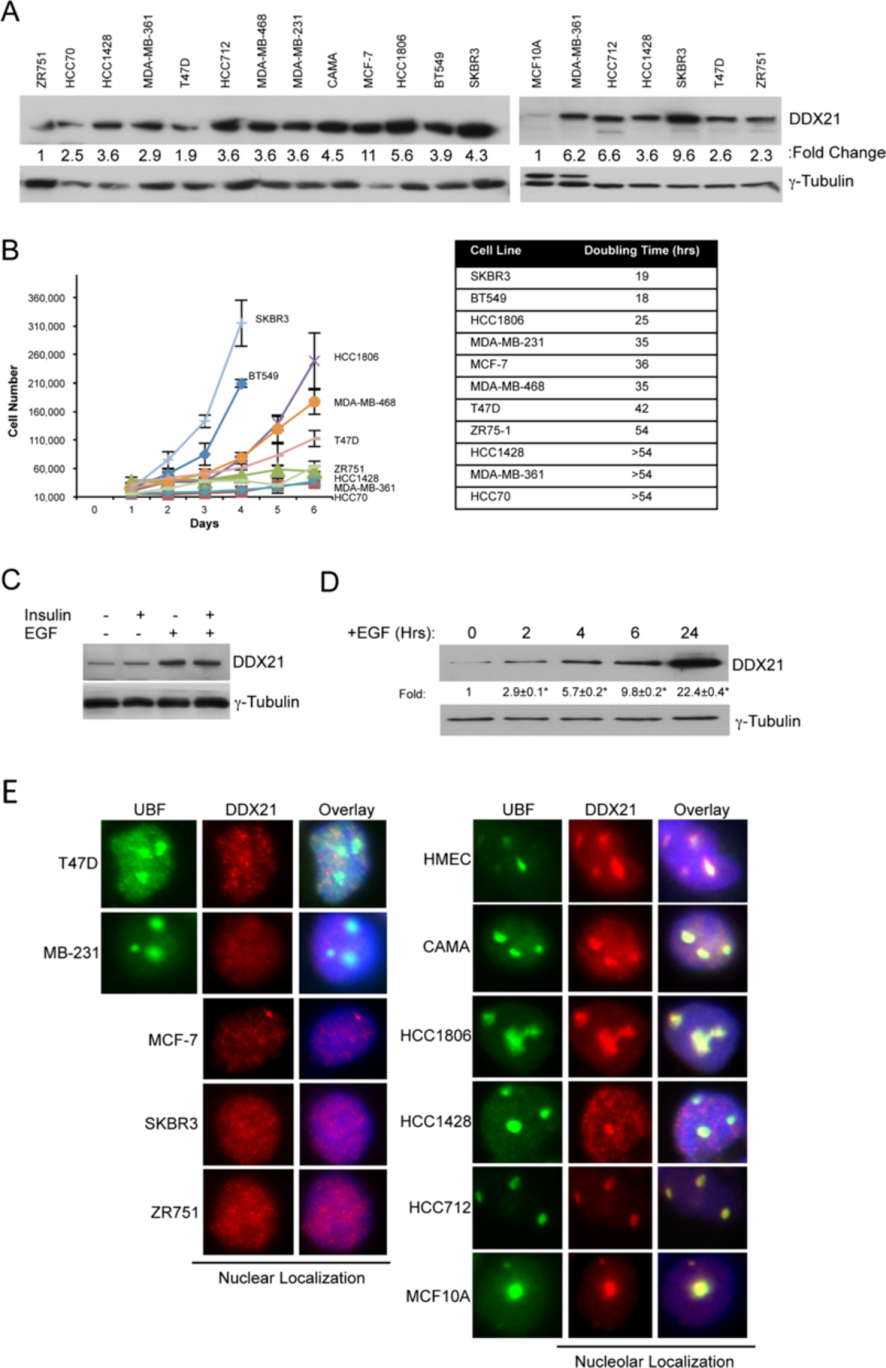
**DDX21 is highly expressed in proliferative breast cancer cell lines and is both nuclear and nucleolar. (A)** A panel of 13 different breast cancer cell lines and nontransformed MCF10A cells were analyzed for DDX21 protein expression by western blot analysis. A total of 50 μg of whole cell extracts was used for analysis. Tubulin was used as a loading control. Quantitation of the signal was made by Image J software and normalized by tubulin levels. **(B)** Equal numbers of the indicated breast cancer cell lines were plated and cell numbers were counted on a daily basis. Bars indicate standard deviation from three separate experiments. Doubling times were estimated based on the growth curves. Doubling time of MDA-MB-231 cells was determined separately. **(C)** MCF10A cells were cultured in regular DMEM media without insulin and EGF supplements for 48 hours. Cells were then fed with DMEM complete media with either insulin alone, EGF alone or both insulin and EGF together for 24 hours. Cells were then harvested and total cell lysates were subjected to western blot analysis with anti-DDX21 antibody and anti-tubulin antibody. **(D)** Above-mentioned growth factor-starved MCF10A cells were treated with 100 ng/ml EGF containing complete media for the indicated time points. Whole cell extracts were subjected to western blot analysis with anti-DDX21 antibody and anti-tubulin antibody. Standard error of the mean is indicated with fold-change. ^*^, *P* <0.05. **(E)** HMECs and breast cancer cell lines were subjected to immunofluorescence analysis with anti-DDX21 to detect cellular localization of DDX21 protein (red). Cell nuclei were demarcated by DAPI (blue) and nucleoli were demarcated by UBF (green). DAPI, 4',6-diamidino-2-phenylindole; DMEM, Dulbecco's modified Eagle's medium; EGF, epithelial growth factor; HMEC, human mammary epithelial cell; UBF, upstream binding factor.

We also analyzed DDX21 protein levels in a panel of established breast cancer cell lines. All of these cancer cells displayed detectable but varying DDX21 protein expression levels (Figure 2A). DDX21 protein levels were significantly higher in cancer cell lines (compared to nontransformed MCF10A cells) that proliferated faster, such as HCC1806, SKBR3, MDA-MB-231 (Figure 2A and B). Conversely, slower proliferating cell lines such as ZR751, HCC70 and HCC1428, expressed much lower DDX21 levels (Figure 2A and B). However, tubulin levels may not serve as the most accurate internal protein control, thus allowing for some differences in our observed expression patterns. In general, and although not absolute, we show that there is a correlation between higher DDX21 levels and overall cell proliferation rate.

In an effort to more accurately assess the connection between proliferation rates and DDX21 expression, we altered the proliferation rate of MCF10A cells through the addition of defined mitogens. MCF10A cells are a nontumorigenic cell line derived from a human mammary gland and its proliferation is dependent on growth factors such as insulin and epidermal growth factor (EGF). Addition of insulin to deprived MCF10A, which only slightly stimulated cell proliferation, resulted in a minimal increase in DDX21 protein expression (Figure 2C). However, addition of EGF, which induced a near maximal proliferation rate, resulted in a significant increase in DDX21 protein expression that was not further affected by insulin (Figure 2C). A time-course analysis following EGF stimulation showed a relatively rapid induction of DDX21 protein expression as early as 2 hours poststimulation (Figure 2D). Thus, DDX21 appears to be a mitogen-induced protein whose expression is indicative of proliferation status *in vitro*. This appears to correlate with a report showing that DDX21 can be regulated by activated MEK signaling [24].

We next performed immunofluorescence analysis to determine the cellular localization of endogenous DDX21 in the same panel of established breast cancer cell lines. Consistent with the immunofluorescence results of primary breast cancer tissues shown in Figure 1A, we found that DDX21 protein was both nuclear and nucleolar. Specifically, DDX21 localized to the nucleoplasm of T47D, MDAMB231, MCF-7, SKBR3 and ZR751 cell lines (Figure 2E), while it localized to nucleoli in CAMA, HCC1806, HCC1428, HCC712 and MDA-MB-468 as well as primary human mammary epithelial cells (HMECs) (Figure 2E).

### DDX21 is essential for breast cancer cell proliferation and survival

**Figure 3:**
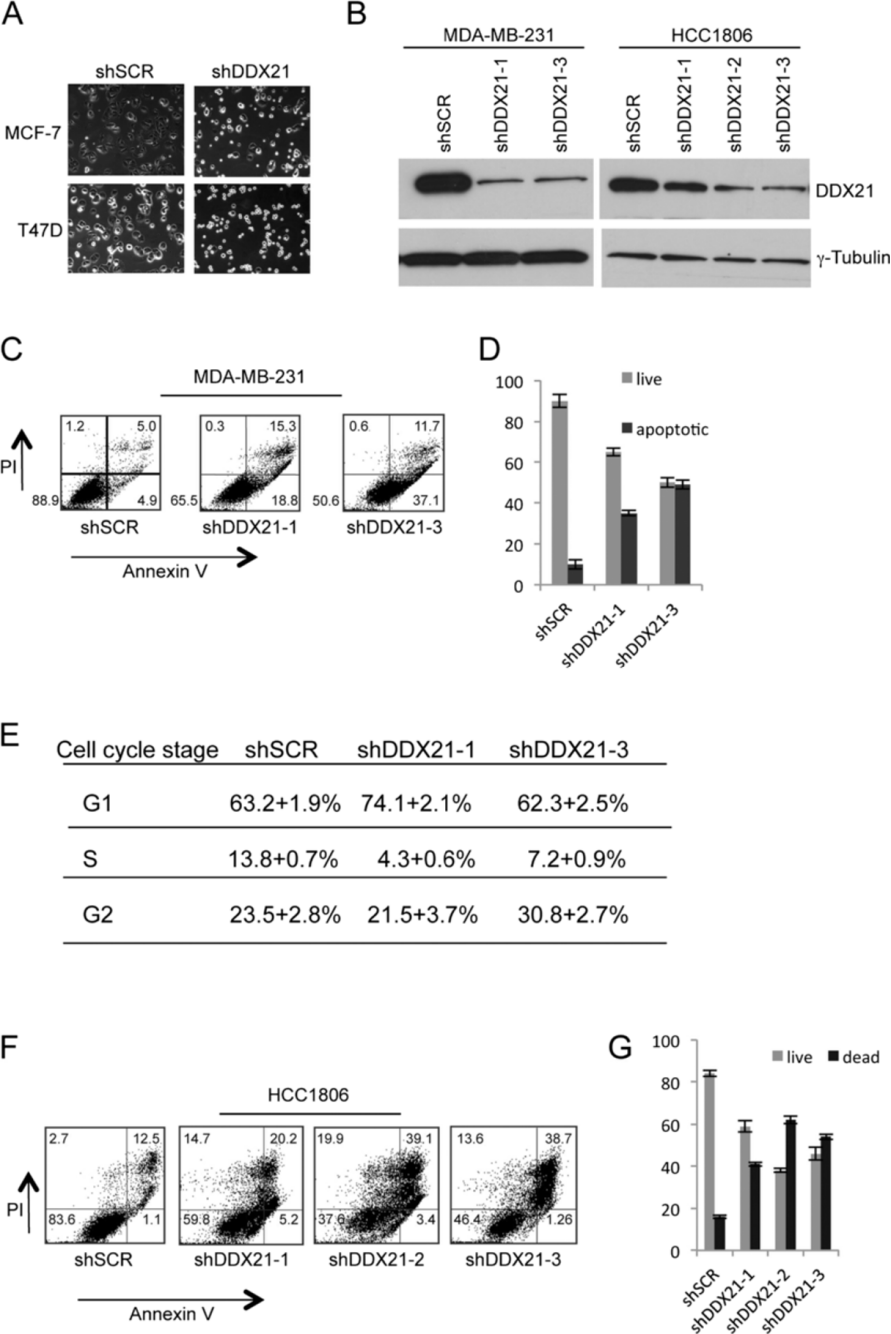
**DDX21 knockdown in multiple breast cancer cell lines induces cell death and cell cycle arrest. (A)** MCF-7 and T47D breast cancer cells were either infected with shSCR or shRNA-DDX21 lentiviruses. Three days postinfection, cells were split and cultured for 48 hours. Typical images for the live culture of the indicated transduced cells are shown. **(B)** MDA-MB-231 cells or HCC1806 cells were either transduced with shSCR or shRNA-DDX21 lentiviruses. Whole cell lysates were subjected to western blot analysis four days and five days postinfection with anti-DDX21 and anti-tubulin antibodies. **(C)** MDA-MB-231 cells were either transduced with shSCR or shRNA-DDX21 lentiviruses. Five days postinfection, cells were then subjected to apoptosis analysis by flow cytometry with Vybrant apoptosis kit after PI labeling of nuclei and FITC labeling of Annexin V. Percentage of live and dead cells are shown in each quadrant. **(D)** Quantitation of live and apoptotic cells were calculated and graphed. **(E)** Above-mentioned MDA-MB-231 cells were subjected to fixation and PI staining before flow cytometry to detect cell cycle distribution. Percentage of cells in each cell cycle phase is presented. **(F)** HCC1806 cells were either transduced with shSCR or shRNA-DDX21 lentiviruses. Five days postinfection, cells were subjected to apoptosis analysis. Population of live and dead cells is marked in each quadrant. **(G)** Quantitation of the live and apoptotic cells is graphed. FITC, fluorescein isothiocyanate.

Given the elevated protein expression of DDX21 in highly proliferative breast cancer cell lines, we sought to determine whether DDX21 was an essential protein for cell proliferation and survival. We utilized multiple shRNAs targeting endogenous human DDX21 to effectively lower DDX21 protein expression. Following successful knockdown of DDX21, we observed a consistent two-to-threefold increase in cell death. Live cell imaging of MCF-7 and T47D cells revealed an increase in apoptotic cells following DDX21 knockdown as evidenced by smaller and more refractive cells (Figure 3A). Reduction of DDX21 with multiple shRNAs in MDAMB231 and HCC1806 cells was confirmed by western blot analysis (Figure 3B). Annexin V staining was used to mark apoptotic cells and results showed a clear increase in apoptotic MDA-MB-231 cells, where DDX21 was primarily nuclear, after DDX21 knockdown (Figure 3C and 3D). Cell cycle analysis revealed a slight, but reproducible, reduction in S phase that coincided with either an increase in G1 or G2/M phases (Figure 3E). Consistent with these results, knockdown of DDX21 in HCC1806 cells, where DDX21 localized primarily to nucleoli, resulted in dramatic increases in cell death. However, Annexin V and PI staining of HCC1806 cells indicated that DDX21 knockdown in HCC1806 cells resulted in necrotic cell death rather than apoptosis (Figure 3F and 3G). Taken together, these results show that elevated DDX21 expression is required in order to maintain the viability and cell cycle progression of highly proliferative breast cancer cells.

### DDX21 is required for the tumorigenicity of breast cancer cells in vitro and in vivo

**Figure 4:**
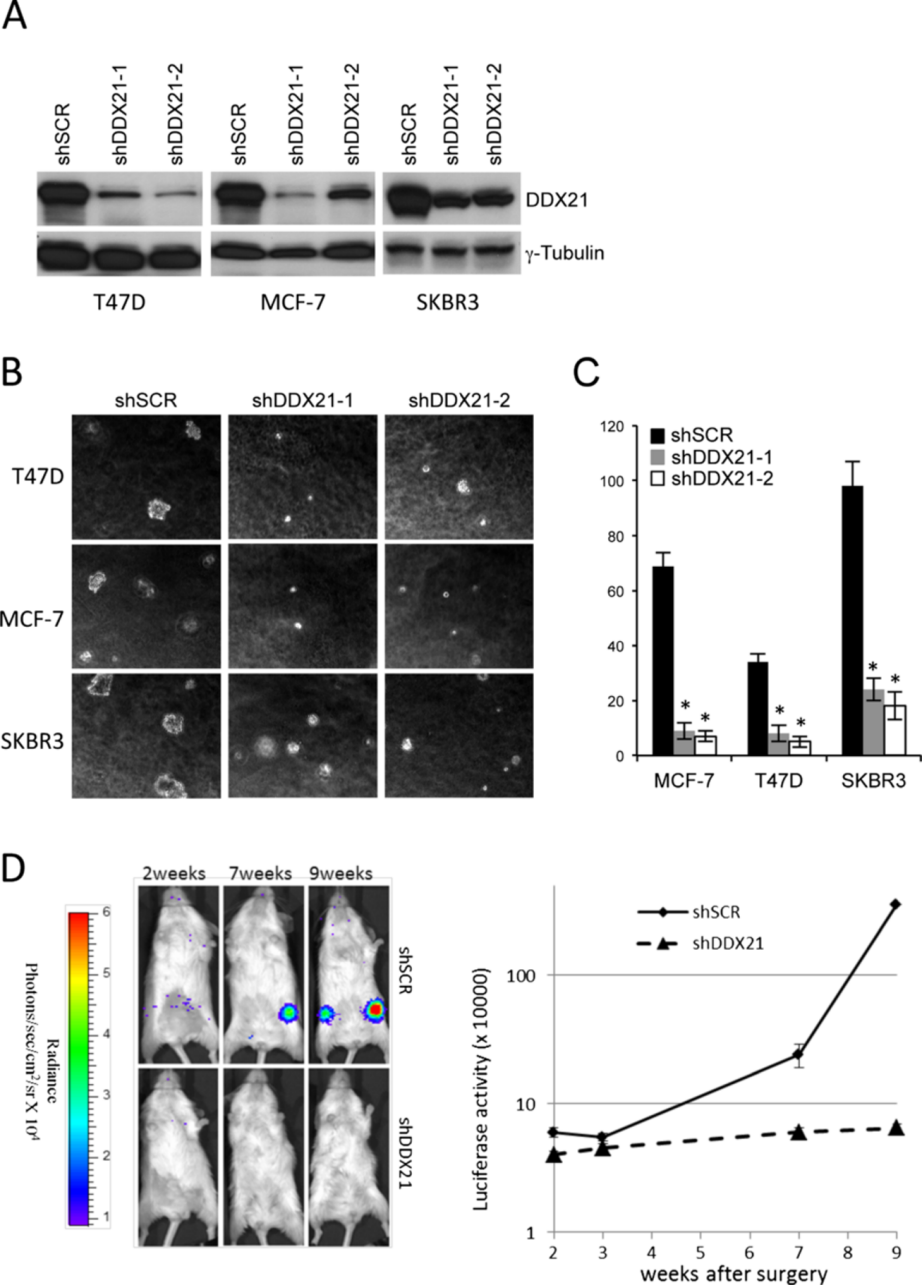
**DDX21 is important for the tumorigenicity of breast cancer cells** ***in vitro*** **and** ***in vivo*** **. (A)** T47D, MCF-7 and SKBR3 cells were transduced with either shSCR or shRNA-DDX21 lentiviruses. DDX21 levels were detected by western blot analysis after lentiviral knockdown. Tubulin was used as a loading control. **(B)** Above-mentioned cells were also subjected to soft agar analysis after plating 1 × 10^4^cells in each 60 mm dish. Typical image is shown after culturing for two weeks. **(C)** Quantitation of colony numbers in the soft agar assay is graphed; error bars were taken from triplicates. **(D)** A group of five SCID mice were injected at the mammary gland on both sides with MDA-MB-231-luciferase reporter cells transduced with either shScrambled or with shDDX21 (50,000 cells per injection). Mice were allowed to recover from the surgery and bioluminescence imaging was performed from two weeks to nine weeks. A representative mouse from each group is shown at the indicated time points. Data presented show the mean × standard deviation from five different mice in each group. SCID, severe combined immunodeficiency.

To assess the long-term effects of DDX21 deficiency on proliferation and tumorigenesis, we utilized multiple shRNAs targeting endogenous human DDX21 to effectively knock down DDX21 protein expression in three highly proliferative breast cancer cell lines (Figure 4A). DDX21 knockdown had a dramatic impact on anchorage-independent cell growth with lower levels of DDX21 resulting in impaired growth in semi-solid soft agar (Figure 4B and C) and indicating that DDX21 might be required for mammary cell tumorigenicity. In addition, we also performed xenograft implants to assess *in vivo* tumorigenesis. Severe combined immunodeficiency (SCID) mice were injected with equal numbers of MDA-MD-231 cells carrying a luciferase reporter and either shScrambled or shDDX21 constructs. At nine weeks post-surgery, the five-shScrambled mice developed steadily increasing signals that were significantly higher than the five-shDDX21 mice (Figure 4D). These results indicate a clear requirement for DDX21 protein expression during breast cancer tumorigenesis *in vitro* and *in vivo*. Our results are also consistent with previous findings that MCF-7 cells (high DDX21) grow significantly faster *in vivo* than ZR-75-1 cells (low DDX21) [25].

### DDX21 modulates AP-1 activity

**Figure 5:**
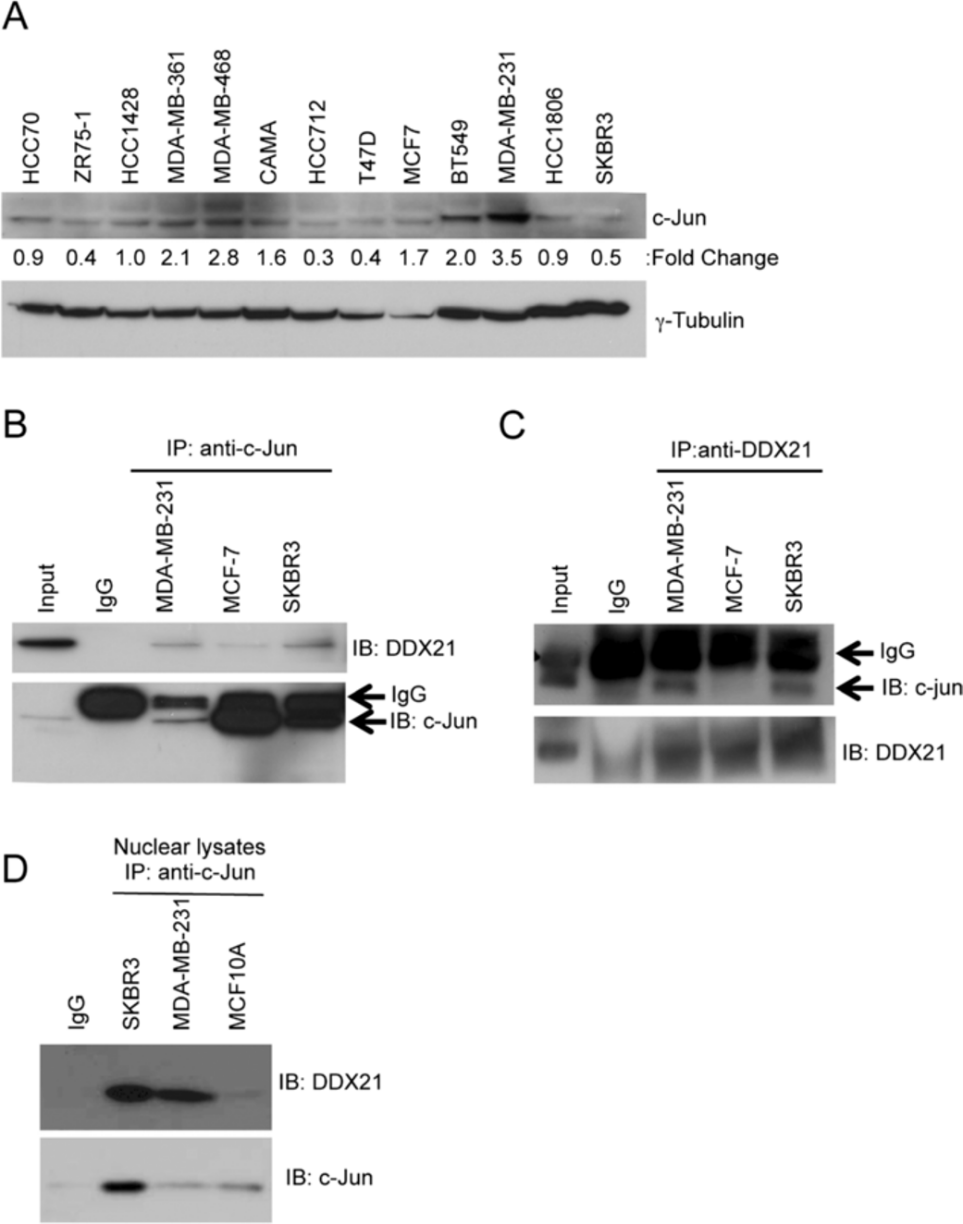
**c-Jun interacts with DDX21 in breast cancer cell lines. (A)** Panel of breast cancer cell lines was screened by western blot analysis with anti-c-Jun antibody. Fifty μg of whole cell lysates were subjected to the analysis and tubulin is used as a loading control. Quantitation of the signal was made by Image J software and normalized by tubulin levels. **(B)** 500 μg of protein lysates were subjected to immunoprecipitation with anti-c-Jun antibody with mouse IgG as a negative control, 25 μg of SKBR3 cell lysate was used as input control. Immune complexes were blotted with anti-DDX21 and anti-c-Jun. **(C)** Reciprocal immunoprecipitation was also performed with the above-mentioned cell lysates. Western blot analysis was performed to confirm the association between c-Jun and DDX21 in breast cancer cell lines. **(D)** c-Jun immunoprecipitation was performed on purified nuclear lysates from the indicated cell lines. Western blot analysis was performed to confirm the association between c-Jun and DDX21.

To begin to understand the molecular function of DDX21, we turned to one of its only known interacting partners, c-Jun [22]. We first examined whether DDX21 interacted with c-Jun in our panel of established breast cancer cell lines. We found that c-Jun was highly expressed in MDA-MB-231 and to a lesser extent in BT549 cells while the remaining lines had very low levels of c-Jun protein expression (Figure 5A). Protein extracts from MDA-MB-231, MCF-7 and SKBR3 cells were subjected to co-immunoprecipitation assays using anti-c-Jun or anti-DDX21 antibodies. We determined that indeed DDX21 associated with c-Jun in each of the cells in reciprocal immunoprecipitations (Figure 5B and C). Additionally, these interactions were enriched when nuclei were isolated prior to performing the immunoprecipitations (Figure 5D), arguing that the interaction of c-Jun and DDX21 in these cells is occurring in the nucleoplasm.

**Figure 6:**
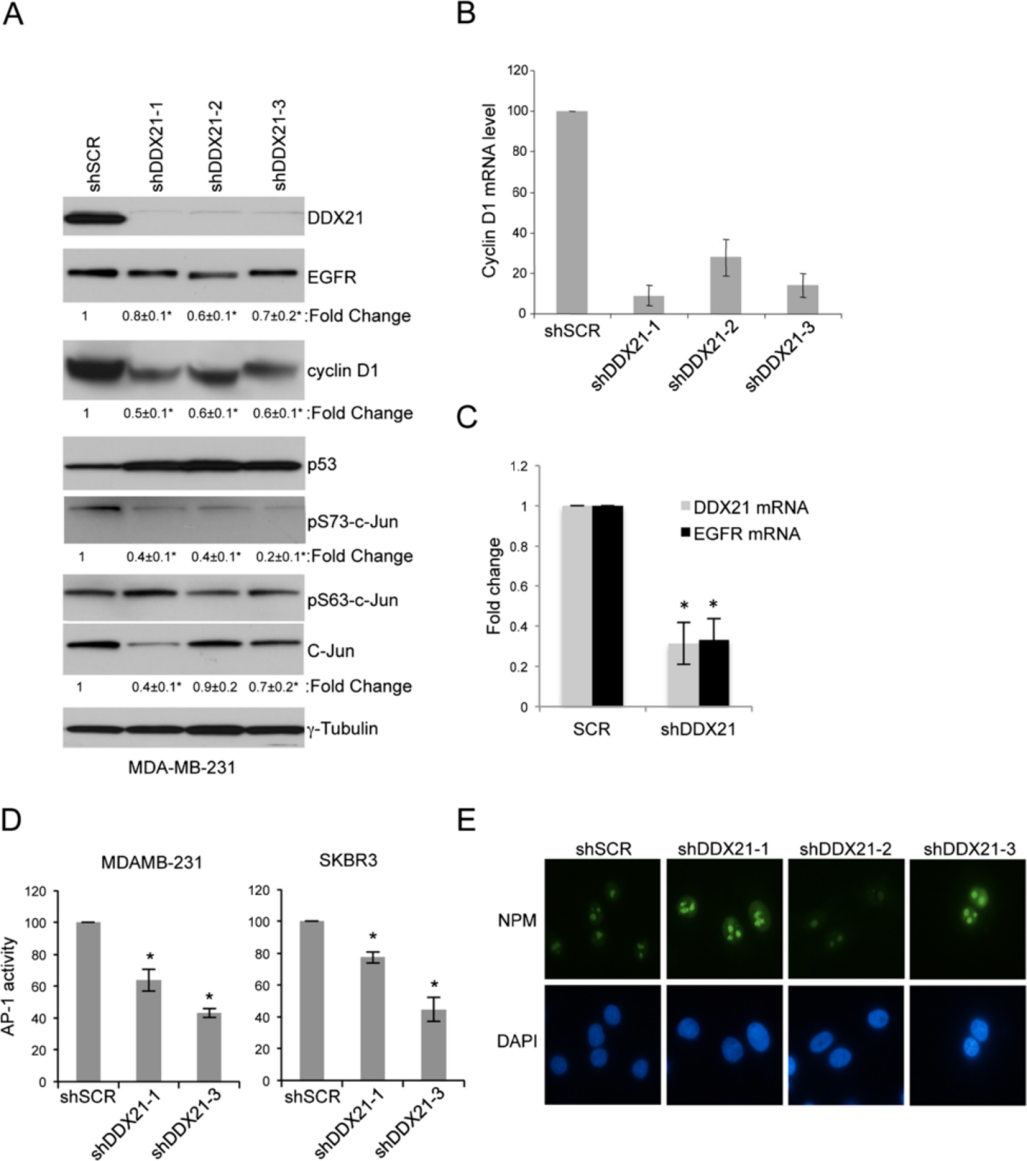
**DDX21 modulates AP-1 activity in MDA-MB-231 cells. (A)** MDA-MB-231 cells were either infected with shSCR or shRNA-DDX21 lentiviruses. Whole cell lysates were subjected to western blot analysis with the indicated antibodies with tubulin as an internal control. Standard error of the mean is indicated with fold-change. ^*^,*P* <0.05. **(B)** RT-PCR analysis was performed for cyclin D1 mRNA levels after normalization with GAPDH mRNA levels. Error bars were taken from three independent experiments. ^*^,*P* <0.001. **(C)** RT-PCR analysis was performed for EGFR mRNA levels after normalization with GAPDH mRNA levels. Error bars were taken from three independent experiments. ^*^,*P* <0.005. **(D)** MDA-MB-231 and SKBR3 cells were infected with AP-1 reporter lentiviruses to detect endogenous AP-1 activity. After verification of AP-1 luciferase activity, these cells were infected with either shSCR or shRNA-DDX21. Two days postinfection, equal numbers of cells were transfected with pGL-*Renilla-*luciferase plasmids. Equal numbers of cells were then analyzed for both firefly and *Renilla* luciferase activity. Data presented is firefly luciferase activity after normalization with *Renilla* luciferase and then further normalized to shSCR control. ^*^,*P* <0.001 (n =3). **(E)** MDA-MB-231 cells infected with shSCR or shRNA-DDX21 lentiviruses were stained with antibodies recognizing NPM and visualized by indirect immunofluorescence. DAPI stain was used to mark nuclei. Images are representative of over 100 cells for each condition. DAPI, 4',6-diamidino-2-phenylindole; EGFR, epithelial growth factor receptor; GAPDH, glyceraldehyde 3-phosphate dehydrogenase; NPM, nucleophosmin; RT-PCR, reverse transcriptase-polymerase chain reaction.

As c-Jun is a critical component of the AP-1 transcription factor, we hypothesized that DDX21 might somehow modulate AP-1 activity in breast cancer cells. To test this hypothesis, we knocked down DDX21 expression in MDA-MB-231 cells and assayed c-Jun activation using antibodies that recognized the phosphorylation of c-Jun on two critical serine residues, Ser63 and Ser73 [12]. DDX21-depleted cells displayed significantly reduced levels of phosphorylated c-Jun on Ser73, the primary site for c-Jun activation, while maintaining consistent total c-Jun levels (Figure 6A).

Importantly, AP-1 activity governs the transcriptional induction of cyclin D1 to promote the enhanced proliferation of established breast cancer cells [1]. Consistent with our connection between DDX21 and c-Jun phosphorylation status, knockdown of DDX21 resulted in a significant decrease in cyclin D1 protein expression (Figure 6A). Similarly, expression of EGFR, another target of AP-1 [4], was also reduced at the protein level by up to 30 to 40% in all three DDX21 knockdown samples (Figure 6A) as well as at the mRNA level (Figure 6C). As expected, cyclin D1 mRNA levels were substantially reduced in DDX21 knockdown cells (Figure 6B), underscoring the transcriptional relationship between DDX21 and known AP-1 targets. To more directly assess the endogenous AP-1 transcriptional activity in DDX21-depleted cells, a firefly luciferase reporter was utilized. We first infected MDA-MB-231 cells with lentiviruses encoding an AP-1-firefly luciferase reporter, and then infected these transduced cells with a second lentivirus encoding the shScramble or shRNA-DDX21. Cells were then transiently transfected with a plasmid encoding a *Renilla* luciferase reporter to control for transfection efficiency. After normalization to *Renilla* luciferase activity, we measured a 40 to 50% reduction in AP-1 activity in DDX21 knockdown cells (Figure 6D). Correspondingly, we observed a reduction in AP-1-directed luciferase activity in SKBR3 cells following DDX21 knockdown (Figure 6D). Taken together, our results demonstrate that DDX21, through its interaction with c-Jun, is required for AP-1 activity and its ability to transcriptionally induce cyclin D1 and EGFR. To rule out the possibility that these findings occurred because of a loss in nucleolar integrity, we stained nucleoli from MDA-MB-231 cells with antibodies recognizing nucleophosmin (NPM). We observed no change in nucleolar staining of NPM when comparing shSCR and shDDX21 cells (Figure 6E), indicating an intact nucleolus in all cells.

### Nucleolar DDX21 is required for rRNA processing in breast cancer cells

**Figure 7:**
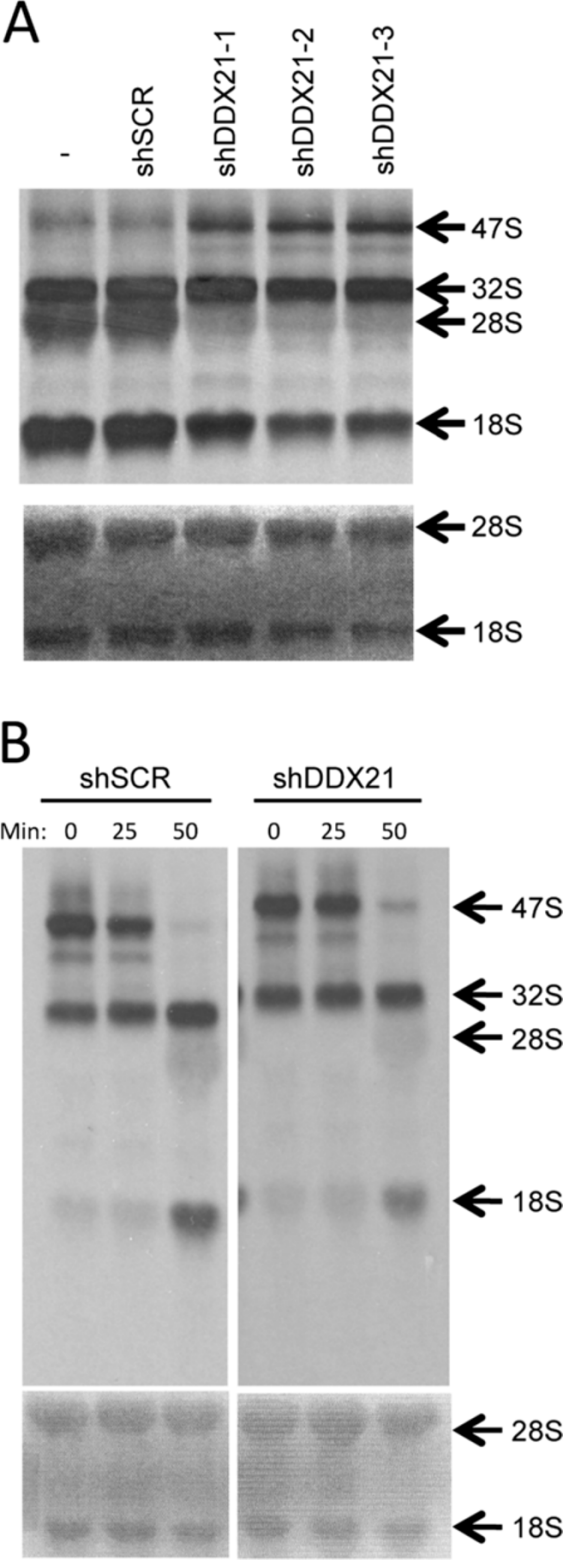
**Nucleolar DDX21 promotes rRNA processing in breast cancer cells. (A)** HCC1806 cells were infected with shSCR or shRNA-DDX21, two days postinfection, cells were pulsed with [^3^H-methyl] methionine and chased for 1 hour. Total RNA was extracted and analyzed for 47S, 32S, 28S and 18S rRNA levels. Methylene blue-stained membrane is also shown at the bottom. **(B)** HCC1806 cells were infected with shSCR or shRNA-DDX21, two days postinfection, cells were pulsed with [^3^H-methyl] methionine and chased for 0, 25 and 50 min. Total RNA was extracted and analyzed for 47S, 32S, 28S and 18S rRNA levels. Methylene blue-stained membrane is also shown at the bottom.

DDX21 was originally identified as a protein that might be involved in rRNA processing based solely on its nucleolar localization. To determine whether DDX21 is a critical component of rRNA processing in our breast cancer system where DDX21 is primarily nucleolar, we first performed an rRNA pulse-chase assay with HCC1806 cells in which DDX21 localizes to the nucleolus. As shown by Figure 7A, DDX21 knockdown caused the accumulation of the rRNA precursor 47S and reduced the total levels of mature 28S and 18S rRNAs, indicating a requirement for DDX21 in the initial processing of the 47S transcript. To determine whether other rRNA processing steps were affected by DDX21 reduction, we followed rRNA processing for 50 minutes. DDX21 knockdown primarily inhibited rRNA processing at the initial 47S processing step and did not result in the accumulation of other downstream intermediates that were not processed properly (Figure 7B), again underscoring the role of DDX21 in the first step 47S rRNA processing.

### DDX21 RNA helicase activity is required for efficient RasV12 cell transformation

**Figure 8:**
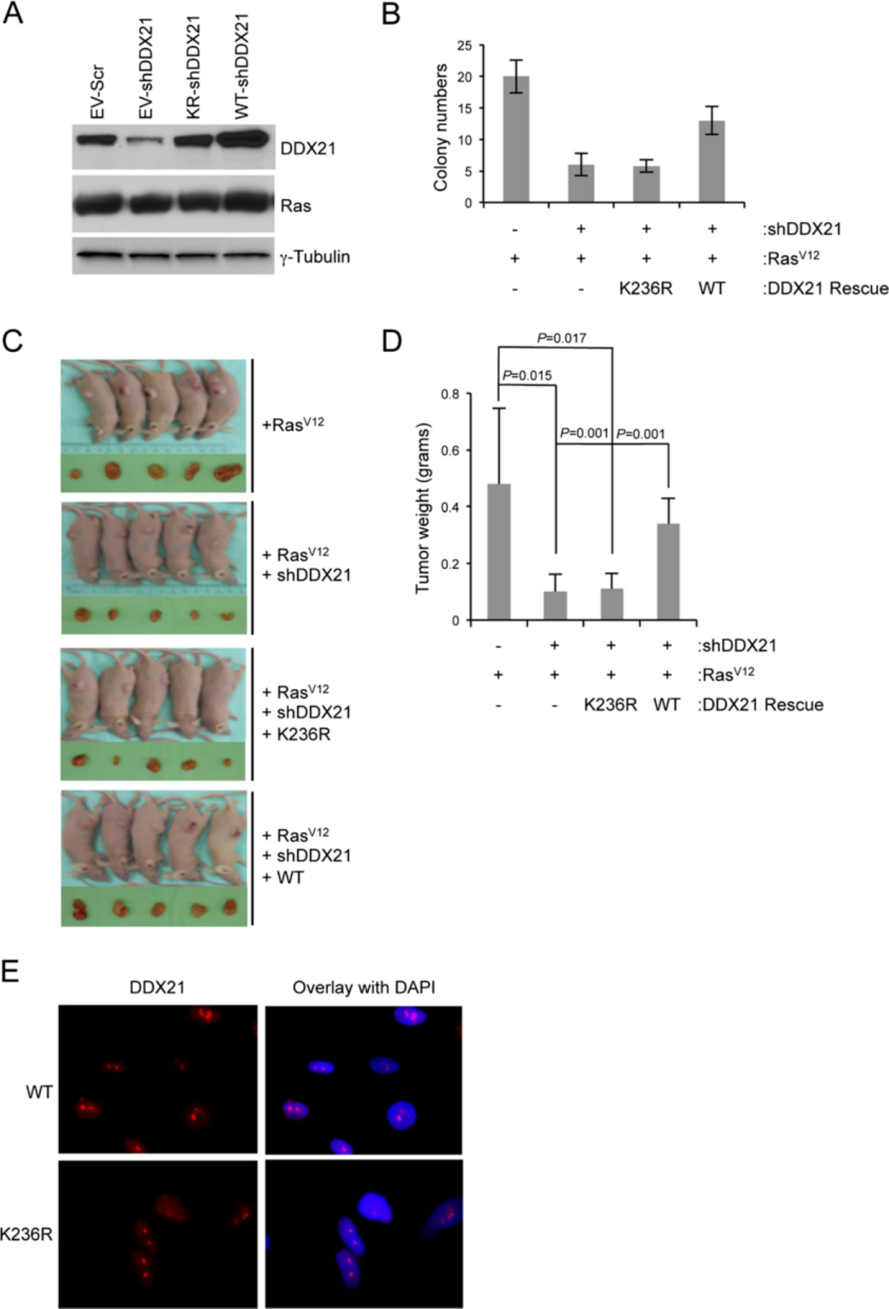
**DDX21 helicase activity is required for enhanced Ras**^**V12**^**transformation. (A)** *Arf*-null MEFs were infected with lentiviruses that encode either wild-type human DDX21 or K236R mutant DDX21 with empty vector as a control. Infected cells were then selected in hygromycin-containing media (500 μg/ml) for three days. Cells were then plated and infected with retrovirus encoding Ras^V12^ or empty vector, and selected in puromycin (4 μg/ml) for two days. Cells were then infected with lentiviruses targeting mouse endogenous DDX21 (shRNA-DDX21) with shSCR as a control. Four days postinfection, whole cell lysate for each sample was then analyzed by western blot with anti-DDX21 antibody, anti-tubulin antibody and anti-Ras antibody. **(B)** For above-mentioned cells, 1 × 10^4^ cells were plated into soft agar for colony-forming assay. Quantitation for the number of colonies was shown. Data presented is the average number of colonies from triplicate plates. Bars indicate standard deviation from each triplicate. ^*^, *P* <0.001. **(C)** Above-mentioned cells (1 × 10^6^ per condition) were injected into the flanks of NUDE mice, after four weeks, mice were sacrificed and tumor were dissected and photographed. **(D)** Tumors from each group were weighed and graphed. **(E)** Above-mentioned cells were fixed and stained with antibodies recognizing DDX21 and visualized by indirect immunofluorescence. DAPI was used to mark nuclei. Images are representative of over 100 cells counted for each condition. DAPI, 4',6-diamidino-2-phenylindole; MEFs, mouse embryonic fibroblasts.

*Ras* is a frequent target for mutation in human cancers. Constitutive activation of Ras through activating mutations, such as Ras^V12^, leads to oncogenic transformation of immortalized mouse fibroblasts [26]. C-Jun has been shown to synergize with oncogenic Ras^V12^ to induce tumor formation [12],[27]. To determine whether DDX21 enhances Ras^V12^'s transforming activity, immortalized (*Arf*-null) MEFs were consecutively infected with lentiviruses encoding wild-type DDX21 and retroviruses encoding oncogenic Ras^V12^, and then shRNA for mouse endogenous DDX21. Conversely, *Arf*-deficient MEFs were infected with lentiviruses encoding a helicase-defective DDX21 mutant at lysine 236. Lysine 236 resides in the ATP-binding motif of DDX21 and mutation of lysine 236 to arginine (K236R) disrupts its ATP-binding activity and severely attenuates its helicase activity. Protein expression levels of DDX21 and Ras are shown in Figure 8A. The tumorigenicity of Ras^V12^ was then analyzed with various levels of DDX21 by soft agar assay. Reduction in endogenous DDX21 resulted in a significant attenuation in soft agar colony formation and wild-type DDX21 overexpression rescued the ability of Ras^V12^-transformed *Arf*-null MEFs to grow in soft agar following endogenous DDX21 knockdown (Figure 8B). However, the helicase dead mutant K236R DDX21 failed to restore the transforming activity of Ras^V12^ (Figure 8B), implying that DDX21 helicase activity was required for effective Ras^V12^ transformation. To determine whether this phenotype could be observed *in vivo*, the above-mentioned cells were injected into the flanks of NUDE mice and the resulting tumors were excised after four weeks. Consistent with our *in vitro* results, only wild-type DDX21 was able to rescue the tumorigenicity of Ras^V12^ (Figure 8C and D), indicating that DDX21 and its RNA helicase activity is pivotal to maintain Ras^V12^ transforming activity *in vivo*. Importantly, nucleolar localization of DDX21 *per se* was not sufficient for its function in promoting cellular transformation as the K236R helicase dead mutant localized to nucleoli (Figure 8E) but failed to rescue transformation.

## Discussion

We report here that DDX21 is overexpressed in a significant number of human breast cancers (up to 25%). Its overexpression preferentially occurs in highly proliferative breast cancer tissues and cells. Apart from its predominant nucleolar localization in many breast cancer cells, we also found that DDX21 localized to the nucleoplasm in a significant number of breast cancer tissues as well as established cancer cell lines. Levels of c-Jun did not appear to regulate the nuclear localization of DDX21, since in MDA-MB-231 cells that had the highest c-Jun levels, DDX21 predominantly localized to the nucleus. This suggests that other regulatory mechanisms might influence the subcellular localization of DDX21. This localization is significant because in these cells we found an association between DDX21 and the c-Jun transcription factor. Originally thought to play a role in rRNA processing due to its nucleolar localization [28], nucleoplasmic localization of DDX21 implies functions outside of rRNA processing. This was recently demonstrated when DDX21 was found to form a complex with DDX1 and DHX36 to sense double-stranded RNA in the cytoplasm of dendrite cells [28]. Our results point to its important function in the nucleus: to promote the activation of c-Jun in epithelial cells. DDX21 sets the activation state of nuclear AP-1 complexes and facilitates its transcriptional activity toward key players in cell proliferation such as cyclin D1. Particularly, in MDA-MB-231 cells where endogenous c-Jun levels are high, DDX21 expression appears to be critical for maintaining AP-1 transcriptional activity. Short hairpins targeting endogenous DDX21 resulted in lowered DDX21 and concomitant decreases in c-Jun Ser73 phosphorylation, a phospho-residue required for AP-1 activation. While it is uncertain how DDX21 might enhance c-Jun phosphorylation through its interaction with c-Jun, lower DDX21 protein expression clearly led to a significant reduction in classic AP-1 transcriptional targets such as cyclin D1 and EGFR.

Large-scale genome sequencing of human cancers has revealed that cancer cells possess large numbers of genetic and epigenetic alterations [29]-[31]. While many of these alterations are likely to be random due to the genomic instability of tumor cells, only a few are regarded as true oncogenes that drive the cancer phenotype [32]. However, increased dependence of cancer cells on normal cellular functions also plays essential roles for maintaining cancer cell phenotype. These genes are often called non-oncogenes in reference to their requirement for cellular transformation while not possessing transforming activities alone [33],[34]. Here, we have shown that DDX21 indeed could fall into this category of genes whose activities are required but not sufficient for cellular transformation. DDX21 expression is high in many primary human breast tumors [19] (and herein) and established breast cancer cell lines. Knockdown of DDX21 resulted in dramatic increases in cell death in numerous breast cancer cell lines. DDX21 expression was required to maintain breast cancer survival and proliferation in an *in vivo* mouse mammary gland. Immortal fibroblasts also required DDX21 expression to be efficiently transformed by oncogenic Ras^V12^ alleles. Together, these results point to an essential role for DDX21 in tumor cell survival, proliferation, and transformation through two independent activities: rRNA processing and c-Jun activation. However, DDX21 alone is not sufficient to transform immortal cells, underscoring its potential role as a non-oncogene. Our results strengthen the concept that has been posited in recent years: that cancer cells rely on non-oncogenes in order to maintain and even enhance their tumorigenic phenotype.

The family of DEAD/DEAH box RNA helicases has recently emerged as a large family (54 members) of multifunctional proteins involved in various steps of RNA and DNA metabolism [35]-[38]. It is becoming apparent that many of these helicases perform functions that are either fundamental in many basic cellular processes such as rRNA synthesis or are more specific in promoting cancer cell growth and proliferation. Even though their detailed molecular mechanisms remain largely undiscovered, our report on DDX21 function underscores the potential importance and diversity of DEAD/DEAH helicases in promoting cancer and certainly warrants a broader evaluation of the activities of this protein family.

## Conclusions

In this study, we found DDX21 overexpressed in highly proliferative primary breast carcinomas. DDX21 was found to localize to the nucleus and nucleolus in a number of breast cancer tissues as well as in numerous established breast cancer cell lines. In multiple breast cancer cell lines, DDX21 protein knockdown triggered cell cycle arrest as well as massive cell death and caused a significant reduction in anchorage-independent cell growth *in vitro* as well as *in vivo*. We found that nuclear DDX21 interacted with the activating protein (AP-1) component, c-Jun. DDX21 was required for c-Jun Ser73 phosphorylation and activation of AP-1 transcriptional activity, while nucleolar DDX21 promoted cell growth by enhancing rRNA processing. Furthermore, DDX21 RNA helicase activity was required for effective Ras^V12^ transformation. These data highlight the importance of DDX21 in breast cancer cell proliferation and transformation through its promotion of AP-1 activity, rRNA processing and its cooperation with known oncogenes.

## Authors' contributions

YZ designed the study, performed the experiments, wrote the manuscript, approved the final version, and agreed to be accountable for all aspects of the work. KCB performed EGFR experiments and nucleolar integrity assays, edited the manuscript, approved the final version, and agreed to be accountable for all aspects of the work. LY helped in the growth curves for most of the cell lines, interpreted data for the work, edited the manuscript, approved the final version, and agreed to be accountable for all aspects of the work. AJS helped to perform NUDE mice injections, interpreted data for the work, contributed to the draft of the manuscript, approved the final version, and agreed to be accountable for all aspects of the work. JDW supervised and designed the study, revised the manuscript, approved the final version, and agreed to be accountable for all aspects of the work. All authors have read and approved the final version of this manuscript.

## Authors' information

YZ is currently an Assistant Professor in South University of Science and Technology of China. AJS is currently a staff scientist in Millipore (St. Louis).

## Additional file

## Electronic supplementary material

Additional file 1: DDX21 expression scores and patient information.(PDF 55 KB)

Below are the links to the authors’ original submitted files for images.

Authors’ original file for figure 1

Authors’ original file for figure 2

Authors’ original file for figure 3

Authors’ original file for figure 4

Authors’ original file for figure 5

Authors’ original file for figure 6

Authors’ original file for figure 7

Authors’ original file for figure 8

## Abbreviations

ARF: alternate reading frame
DAPI: 4',6-diamidino-2-phenylindole
DMEM: Dulbecco's modified Eagle's medium
EGF(R): epithelial growth factor (receptor)
ER: estrogen receptor
FACS: fluorescence-activated cell sorting
FBS: fetal bovine serum
FITC: fluorescein isothiocyanate
GAPDH: glyceraldehyde 3-phosphate dehydrogenase
Her-2: human epidermal growth factor receptor 2
HMEC: human mammary epithelial cell
MEF: mouse embryonic fibroblast
NPM: nucleophosmin
ORF: open reading frame
PBS: phosphate-buffered saline
PR: progesterone receptor
PVDF: polyvinylidene fluoride
qRT-PCR: quantitative reverse transcriptase-polymerase chain reaction
SCID: severe combined immunodeficiency
shRNA: short hairpin RNA
UBF: upstream binding factor
