# Supplementary material for: Elevated DDX21 regulates c-Jun activity and rRNA processing in human breast cancers
Source: Breast Cancer Res. 2014 Sep 28;16:449. doi: 10.1186/s13058-014-0449-z (PMC4303128; doi:10.1186/s13058-014-0449-z)
Supplement: Supplementary file 1 — Additional file 1: DDX21 expression scores and patient information.(PDF 55 KB) [file 13058_2014_449_MOESM1_ESM.pdf]

# Additional File 1: DDX21 expression scores and patient information

(DDX21 scores based on percentage of DDX21 positive and signal strength: 0-25%=1; 25-50%=2; 50-100%=3 weak=1; moderate=2; strong=3, added together)

| CASE NO. | ER | PR | p53 | Ki67 | Her2 | DDX21 Localization | DDX21 Score |
|---|---|---|---|---|---|---|---|
| 1 | - | - | - | +++, 90% | - | Nucleolar | 5 |
| 2 | - | - | +++, 100% | +++, 20% | +++ | Nucleolar | 3 |
| 3 | +++ | ++, 2% | +, 5% | ++, 50% | ++ | Nucleolar | 4 |
| 4 | - | - | - | +++, 50% | ++ | Nuclear | 4 |
| 5 | +++, 80% | +,0.5% | ++, 50% | +++, 20% | +++ | Nucleolar | 3 |
| 6 | +, 60% | - | - | +++, 15% | +++ | Nucleolar | 3 |
| 7 | - | - | +, 5% | +++, 80% | +++ | Nucleolar | 5 |
| 8 | +++, 90% | +++, 50% | - | +++, 60% | +++ | Nuclear | 5 |
| 9 | - | - | +++, 100% | +++, 90% | +++ | Nucleolar | 5 |
| 10 | - | +++,20% | +,1% | +++, 20% | +++ | Nucleolar | 4 |
| 11 | - | - | +++, 99% | +++, 90% | ++ | Nuclear | 5 |
| 12 | +, 50% | - | +,50% | +++,95% | ++ | Nucleolar | 5 |
| 13 | ++, 80% | +++, 90% | +++, 40% | +++, 85% | +++ | Nucleolar | 5 |
| 14 | - | - | - | ++, 20% | +++ | Nucleolar | 3 |
| 15 | - | - | +++, 99% | +++, 90% | - | Nuclear | 5 |
| 16 | - | - | - | +++, 60% | - | Nucleolar | 4 |
| 17 | - | - | +,1% | +++, 100% | ++ | Nucleolar | 6 |
| 18 | - | - | ++, 80% | +, 8% | +++ | Nuclear | 4 |
