# Supplementary figures and images for: Elevated DDX21 regulates c-Jun activity and rRNA processing in human breast cancers
Source: Breast Cancer Res. 2014 Sep 28;16:449. doi: 10.1186/s13058-014-0449-z (PMC4303128; doi:10.1186/s13058-014-0449-z)

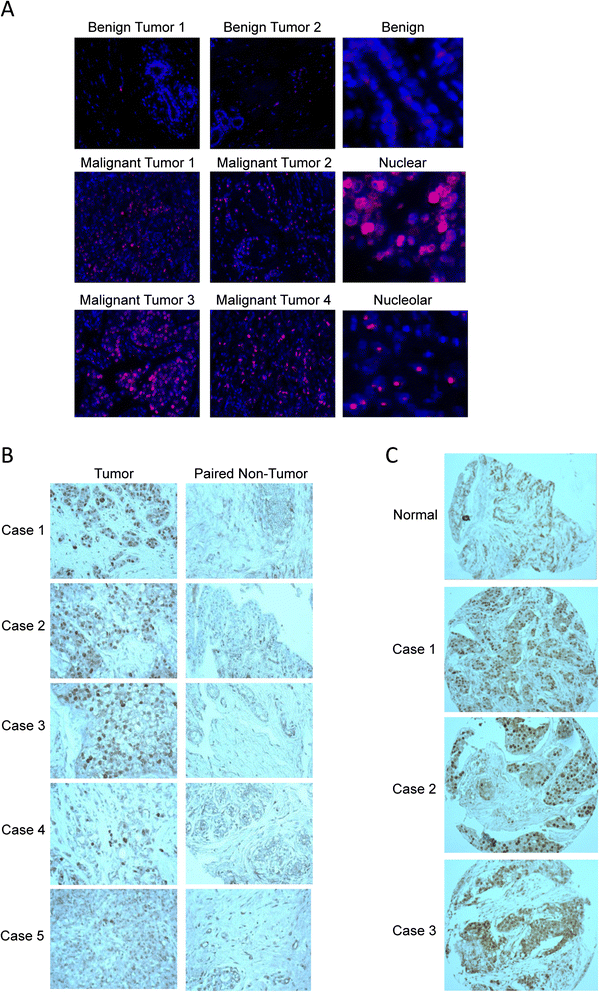

Supplement: Supplementary file 2 — Authors’ original file for figure 1 [file 13058_2014_449_MOESM2_ESM.gif]

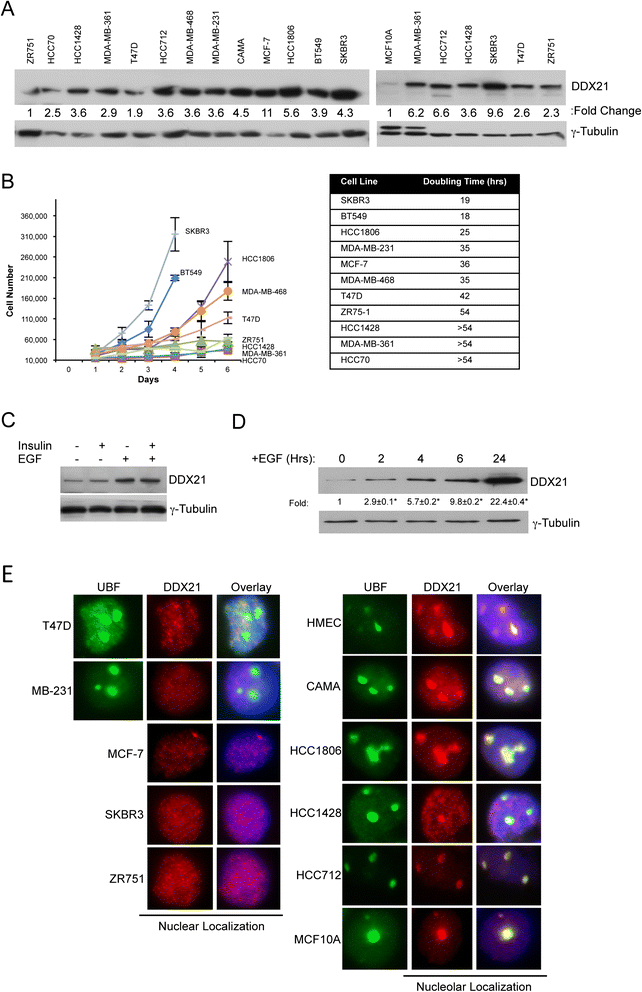

Supplement: Supplementary file 3 — Authors’ original file for figure 2 [file 13058_2014_449_MOESM3_ESM.gif]

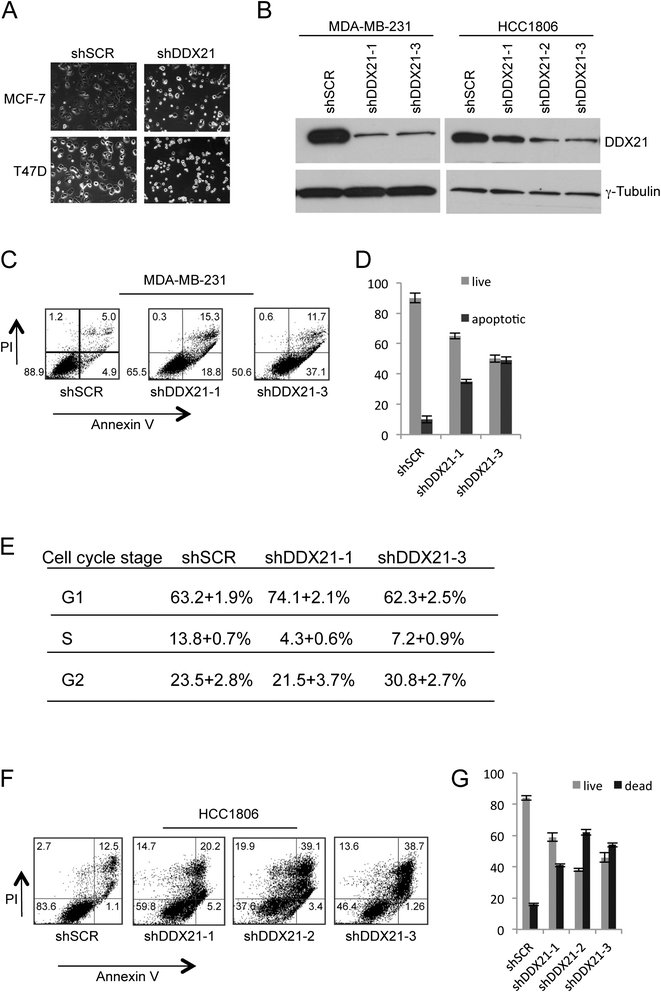

Supplement: Supplementary file 4 — Authors’ original file for figure 3 [file 13058_2014_449_MOESM4_ESM.gif]

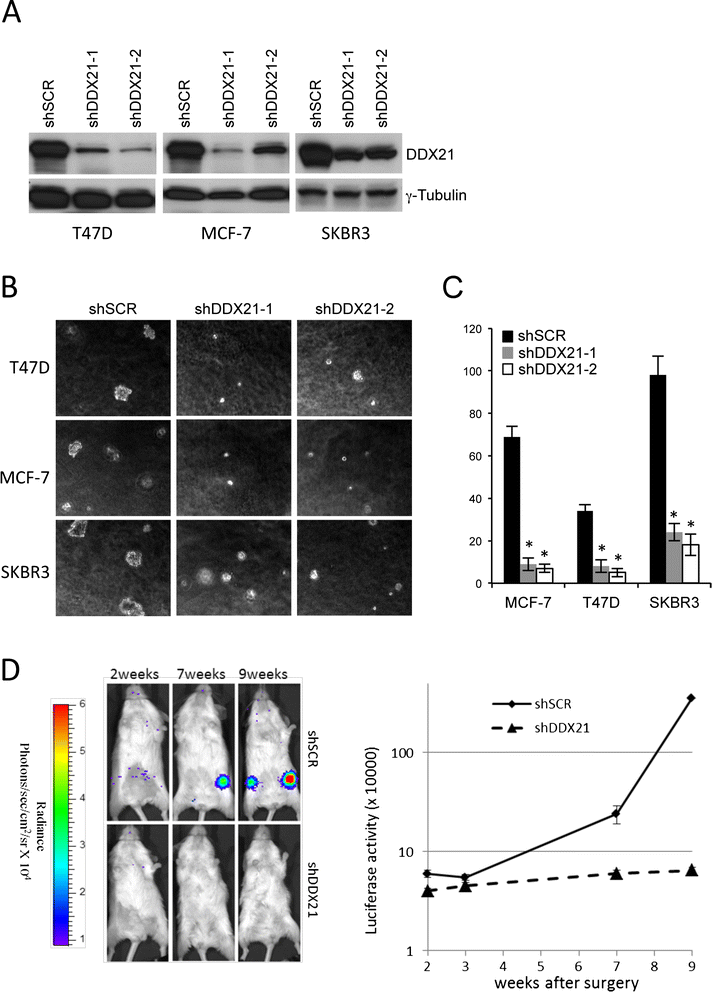

Supplement: Supplementary file 5 — Authors’ original file for figure 4 [file 13058_2014_449_MOESM5_ESM.gif]

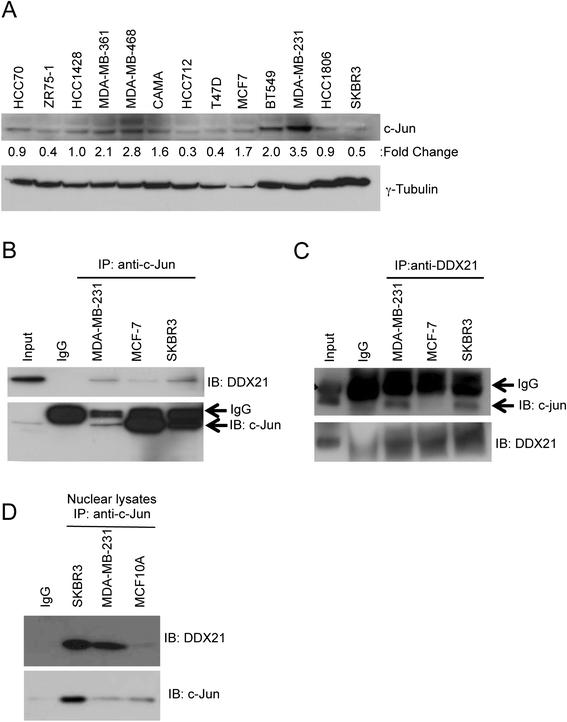

Supplement: Supplementary file 6 — Authors’ original file for figure 5 [file 13058_2014_449_MOESM6_ESM.gif]

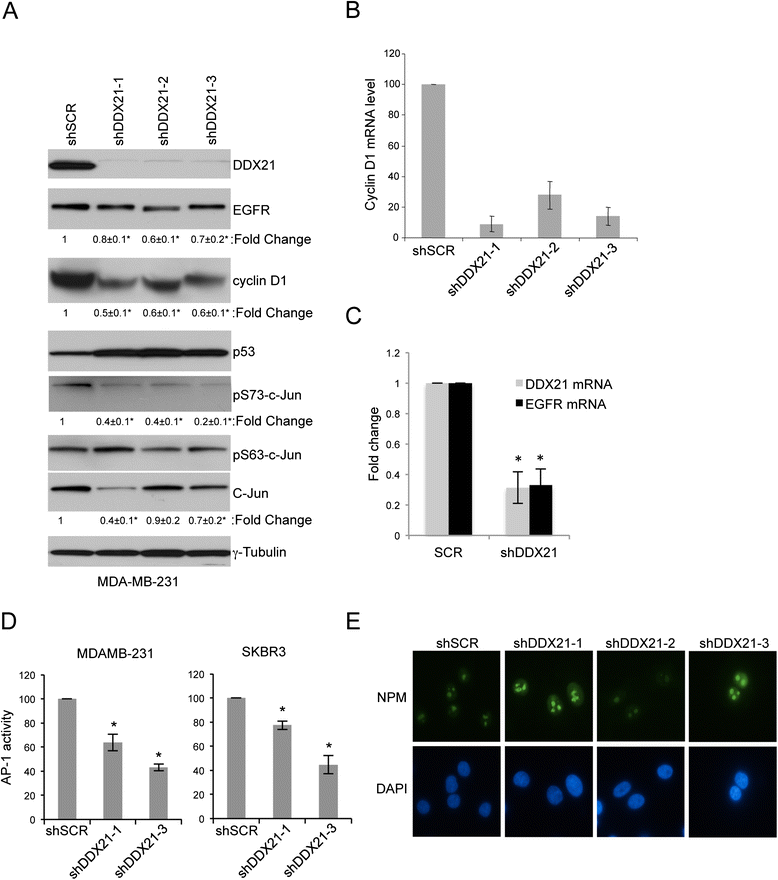

Supplement: Supplementary file 7 — Authors’ original file for figure 6 [file 13058_2014_449_MOESM7_ESM.gif]

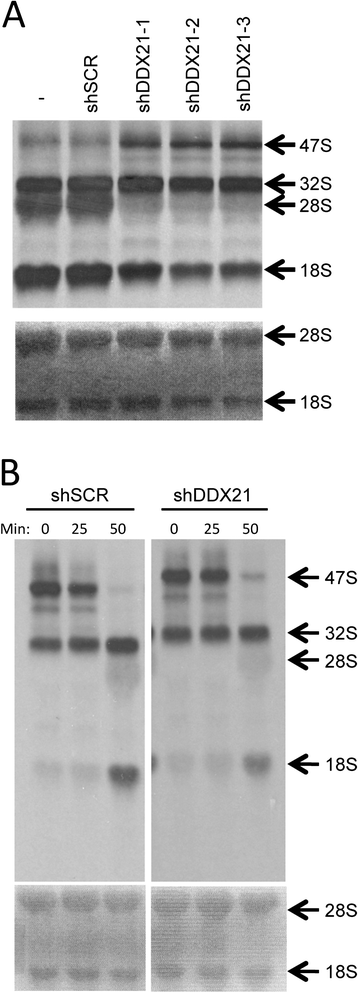

Supplement: Supplementary file 8 — Authors’ original file for figure 7 [file 13058_2014_449_MOESM8_ESM.gif]

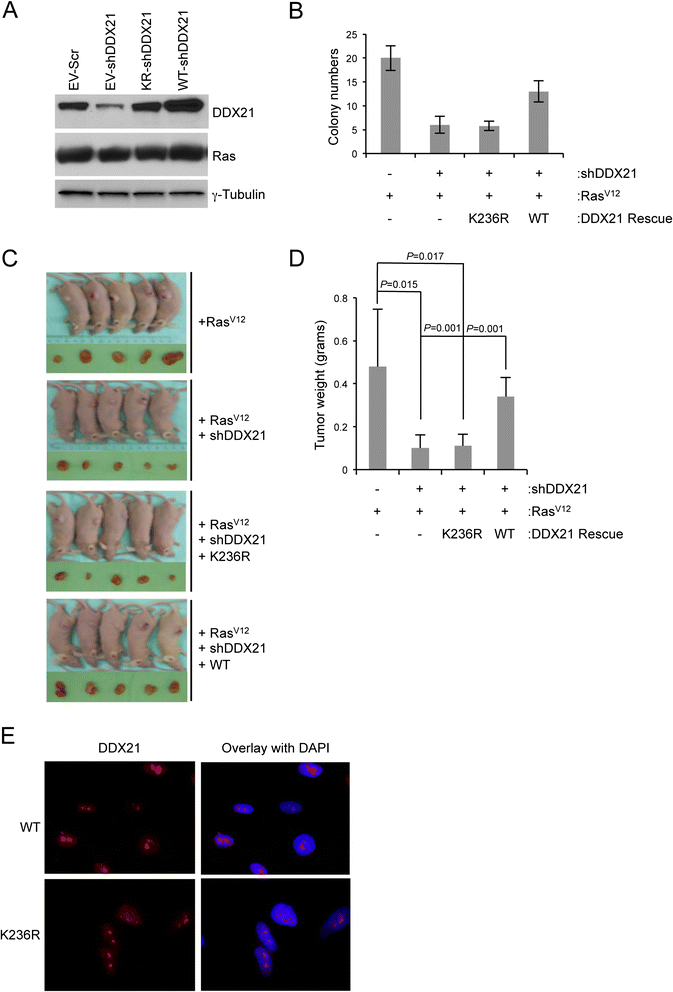

Supplement: Supplementary file 9 — Authors’ original file for figure 8 [file 13058_2014_449_MOESM9_ESM.gif]
